# How to prophylactically alleviate postembolization syndrome following transarterial chemoembolization? Protocol of a double blinded, randomized, placebo-controlled trial

**DOI:** 10.1097/MD.0000000000025360

**Published:** 2021-04-09

**Authors:** Yi Pan, Rui Chang, Zhonglin He, Ming Hong

**Affiliations:** a The Ninth People's Hospital of Chongqing; b Chongqing Orthopedic Hospital of Traditional Chinese Medicine, Chongqing, Chongqing City, China.

**Keywords:** Chinese herbs, hepatocellular carcinoma, postembolization, transarterial chemoembolization

## Abstract

**Introduction:**

Hepatocellular carcinoma (HCC) is the most common type of primary liver cancer, and most patients in China are diagnosed at the intermediate or later stages, which is not suitable for the first line therapies. Transarterial chemoembolization (TACE) is a commonly selected therapeutic option for intermediate and later stage HCC in China, but patients often suffer from postembolization syndrome (PES), manifesting as fever, liver area pain, nausea, vomiting, paralyzed intestinal obstruction, and abdominal pain after TACE. We try to conduct a double blinded, randomized, placebo-controlled clinical trial to observe whether Chaihu Guizhi decoction (CGD), a classic traditional Chinese formula, could prophylactically alleviate the incidence of PES in HCC patients after TACE.

**Methods:**

Patients will be randomly assigned sequentially in a 1:1 ratio by using preformed randomization envelopes. After TACE procedures, patients in the treatment group will be administrated with Chinese herbal formula CGD, and patients in the control group with CGD simulations, twice a day, continuously for 7 days. The outcomes are the incidence of PES hospitalization and, complications. SPSS version 22 (IBM, Chicago, IL) will be used for the data, and a *P* < .05 will be considered statistically significant.

**Conclusions:**

The findings will explore the prophylactic effect of CGD in alleviating the incidence of PES following TACE in HCC patients.

**Trial registration:**

OSF Registration number: DOI 10.17605/OSF.IO/FKRSN

## 1 Introduction

Hepatocellular carcinoma (HCC) is the most common type of primary liver cancer and it's the third most common cause of cancer-related death worldwide.^[1,2]^ The incidence is highest in China because it's strongly influenced by chronic hepatitis B and C infection.^[3,4]^ The best therapeutic approach for HCC is according to stages, and patients at an earlier stage may benefit from resection, transplantation, or ablation. Unfortunately, most patients in China are diagnosed at the intermediate or later stages, which is not suitable for the first line therapies.

Currently, transarterial chemoembolization (TACE)^[5,6]^ is the most commonly selected therapeutic option for intermediate and later stage HCC in China, and it has become a main clinical application to control the development of HCC to prolong patient's life. However, because of the systemic inflammatory response caused by cytolysis, or necrosis of hepatocytes, with or without the chemotherapy drug toxicity, as many as 60% to 80% of patients^[7,8]^ often suffer from postembolization syndrome (PES), the most common adverse events of TACE, manifesting as fever, liver area pain, nausea, vomiting, paralyzed intestinal obstruction, and abdominal pain, according to many reports.^[7,8]^ Though it is usually considered to be self-limiting, PES could last for 2 weeks.^[9,10]^ In addition, PES was reported to increase the hospitalization, and decreased quality of life in patients.^[11]^

The administration of analgesics, antiemetic, and antipyretics^[11]^ could be applied for PES with certain effects, but how to prevent the occurrence of PES is more important. In China, traditional Chinese herbs are often used for the treatment and prevention of PES following TACE in HCC patients, and they were reported to prevent PES after TACE and prolong overall survival of unresectable HCC patients.^[12]^ However, there is still a lack of high quality evidence to prove the efficacy. Here we try to conduct a double blinded, randomized, placebo-controlled clinical trial to observe whether Chaihu Guizhi decoction (CGD), a classic traditional Chinese formula, could prophylactically alleviate the incidence of PES in HCC patients after TACE.

## 2 Methods

### 2.1 Study design

**Figure 1:**
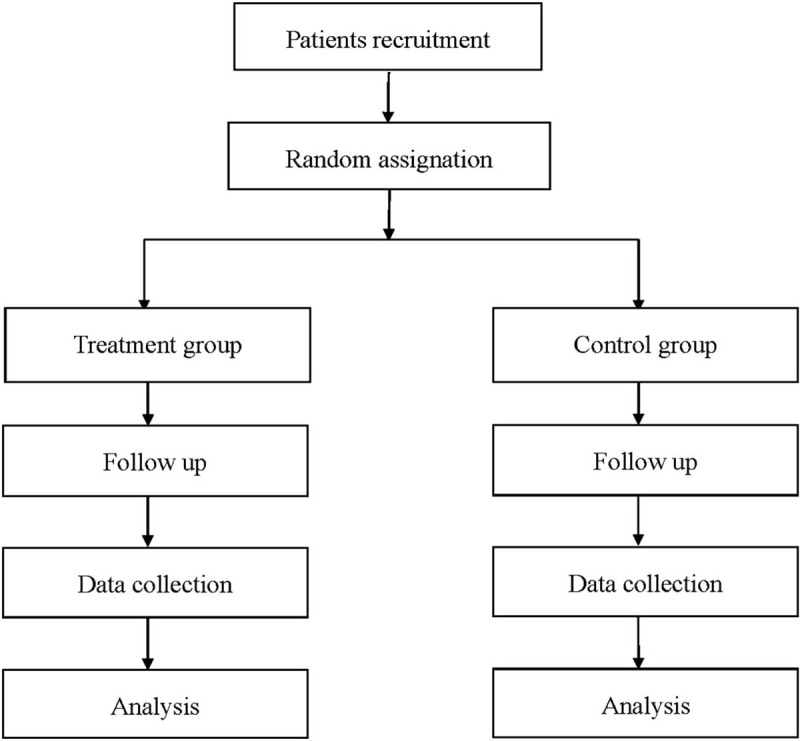
Flow diagram of the study.

This is a prospective, double blinded, randomized, placebo-controlled trial conducted in the Ninth People's Hospital of Chongqing and Chongqing Orthopedic Hospital of Traditional Chinese Medicine from April 1, 2021 to March 31, 2022, (registration number: DOI 10.17605/OSF.IO/FKRSN). The study design is in Fig. 1. The protocol has been approved by the Health Research Ethics Board in the Ninth People's Hospital of Chongqing, and the procedure will be carried out in accordance with the Declaration of Helsinki.

### 2.2 Participants

#### 2.2.1 Recruitment

Patients with unresectable HCC and willing to perform TACE in hospital will be assessed for eligibility. Before randomization, all patients will sign a written informed consent, and they can freely choose whether to continue the trial at any time.

#### 2.2.2 Inclusion and exclusion criteria

The inclusion criteria are as follows: aged between 18 and 80 years old; with unresectable HCC and willing to perform TACE; Child–Pugh class A disease; serum creatinine level lower than 1.5 mg/dL, and the exclusion criteria are as follows: concurrent underlying cardiac, respiratory, or renal disease; other concurrent primary malignancy, allergic to Chinese herbs.

### 2.3 Randomization and blinding

Patients will be randomly assigned sequentially in a 1:1 ratio by using preformed randomization envelopes with SAS software package V.9.4 (Cary, NC). The physicians, patients, data collectors, and outcome assessors will be blinded to the allocation. To maintain blinding, CGD will be provided as granules in the treatment group, and CGD simulation also as granules in the control group.

### 2.4 Interventions

#### 2.4.1 TACE procedures

TACE procedures will be performed in patients of both group as follows: after percutaneous puncture using Seldinger technique, insert the 5F femoral artery sheath and RH catheter, confirm tumor enhancement, and tumor-feeding arteries with angiography, selectively catheterize arteries, inject Oxaliplatin 100 to 150 mg, followed by embolization with Doxorubicin 20 mg and Lipiodol 20 mL and 1-mm-diameter absorbable gelatin sponge particles.

#### 2.4.2 Treatment after TACE

After the TACE, patients in the treatment group will be administrated with Chinese herbal formula CGD, composed of *Ramulus cinnamomi* (Guizhi) 10 g, *Scutellaria baicalensis* (Huangqin) 10 g, *Ginseng* (Renshen) 15 g, *Licorice* (Gancao) 10 g, *Pinellia pinellia* (Banxia) 5 g, *Radix paeoniae alba* (Baishao) 15 g, *Jujube* (Zao) 10 g, *Ginger* (Shengjiang) 10 g, and *Radix bupleuri* (Chaihu) 30 g. The herbs will be provided as concentrate-granules in one package and quality controlled by Beijing Tcmages Pharmaceutical CO., Ltd. Patients will orally take 100 mL of the CGD decoction twice a day, continuously for 7 days.

Patients in the control group will be administrated with CGD simulations twice a day, continuously for 7 days. The simulations are composed of amylum, which is the same with CGD granules in appearance, taste, dosage, and administration methods. If PES in both groups cannot be alleviated after taking CGD or CGD-simulations, patients will be treated with antiemetic for nausea and vomiting, analgesics for pain, and antipyretics for fever.

### 2.5 Outcome measures

The primary outcome is the incidence of PES manifesting as one or several of the following symptoms: nausea, vomiting, fever, paralyzed intestinal obstruction, and abdominal pain at 1d, 3d, 7d, and 14d. The secondary outcomes are length of hospitalization after the treatment, and the incidence of complications at d 14. All related complications and adverse events (AEs) will also be recorded.

### 2.6 Sample size

It was previously reported that the incidence of PES is approximately 70%,^[13]^ and our preliminary study showed that the incidence was 30% after taking CGD. Therefore, the sample size will be 25 in each group, taking a 2-sided *t* test with α = 5% and 1–β = 80%, and a potential dropout of 10%.

### 2.7 Statistical methods

Continuous data will be expressed as mean ± standard deviation, and categorical data as percentages (%). The difference of continuous data will be analyzed by Student *t* test, Wilcoxon rank-sum test, or Manne–Whitney *U* test, and Chi-squared test or Fisher exact test for categorical data. SPSS version 22 (IBM, Chicago, IL) will be used for the data, and a *P* < .05 will be considered statistically significant.

## 3 Discussion

Hepatocellular carcinoma shares a large proportion of liver cancer patients. Many patients are already in the intermediate or later stages when they are diagnosed, and the first line therapies, resection, or transplantation, are not suitable. Globally, with the continuous development of interventional therapy, TACE is the preferential treatment for unresectable HCC and an alternative treatment for intermediate HCC.^[14–16]^ However, due to the adverse effects of chemotherapy drugs and the damage to the immune function, most patients are accompanied by PES after TACE,^[17]^ such as abdominal pain, nausea and vomiting, and fever, which seriously lower the prognosis and quality of life in HCC patients.

In China, traditional Chinese herbal formulas are widely used for alleviating pain, nausea and vomiting, and fever in many diseases. Chaihu Guizhi Decoction (CGD) is a classic herbal formulas^[18]^ and has been applied for nausea, vomiting, and fever for thousands years. In our group, we have tried to administrate the CGD for HCC patients performing TACE to alleviate PES, and the preliminary results showed that the prophylactic administration of CGD might function in alleviating the incidence of PES following TACE. Therefore, we try to perform a double blind, randomized, placebo-controlled clinical trial to explore the efficacy and safety of CGD in prophylactically alleviating the incidence of PES following TACE in HCC patients.

There are several limitations in the study. First, it will be performed in 2 centers. Second, the placebo-control design may lead to uncontrollable dropout. Third, all chemoembolization procedures are conventional with doxorubicin.

## Author contributions

**Data curation:** Yi Pan, Rui Chang.

**Funding acquisition:** Rui Chang.

**Funding support**: Rui Chang.

**Investigation:** Zhonglin He, Ming Hong.

**Resources:** Zhonglin He.

**Software:** Yi Pan.

**Supervision:** Zhonglin He, Ming Hong.

**Writing – original draft:** Yi Pan, Rui Chang.

**Writing – review & editing:** Yi Pan, Rui Chang.
